# Transparent Metasurface for Generating Microwave Vortex Beams with Cross-Polarization Conversion

**DOI:** 10.3390/ma11122448

**Published:** 2018-12-03

**Authors:** Hongyu Shi, Luyi Wang, Mengran Zhao, Juan Chen, Anxue Zhang, Zhuo Xu

**Affiliations:** 1 School of Electronic and Information Engineering, Xi’an Jiaotong University, Xi’an 710049, China; honyo.shi1987@gmail.com (H.S.); bigcrash@stu.xjtu.edu.cn (L.W.); zmr1993@stu.xjtu.edu.cn (M.Z.); anxuezhang@xjtu.edu.cn (A.Z.); 2 Xi’an Jiaotong University Shenzhen Research School, Shenzhen 518057, China; 3 Electronic Materials Research Laboratory, Key Laboratory of the Ministry of Education, Xi’an Jiaotong University, Xi’an 710049, China; xuzhuo@xjtu.edu.cn

**Keywords:** vortex beam, polarization conversion, orbital angular momentum

## Abstract

In this paper, metasurfaces with both cross-polarization conversion and vortex beam-generating are proposed. The proposed finite metasurface designs are able to change the polarization of incident electromagnetic (EM) waves to its cross-polarization. In addition, they also can modulate the incidences into beams carrying orbital angular momentum (OAM) with different orders (l=+1,l=+2,l=−1 and l=−2) by applying corresponding transmission phase distribution schemes on the metasurface aperture. The generated vortex beams are at 5.14 GHz. The transmission loss is lower than 0.5 dB while the co-polarization level is −10 dB compared to the cross-polarization level. The measurement results confirmed the simulation results and verified the properties of the proposed designs.

## 1. Introduction

The orbital angular momentum of electromagnetic waves has been explored in recent years for its potential applications in wireless communications [1,2,3] and imaging [4,5]. EM wave carrying orbital angular momentum has a helical wavefront and an amplitude singularity in the propagating direction. The helical wavefront can be expressed by the term exp(ilΦ), where Φ is the azimuthal angle and *l* is the topological charge. The topological charge corresponds to the OAM mode and, theoretically, the OAM mode is vast.

The OAM in EM waves is typically generated using techniques like spiral phase plates [6,7], spiral reflectors [8], antennas [9,10,11], dieletric resonators [12], computer-induced holograms [13], transformation electromegnetics [14] and metasurfaces [15,16]. The common idea in these techniques is to introduce the desired phase distribution on the radiation aperture. The spiral phase plate method gives the incident wave different phase retardation according to the term exp(ilΦ) by modulating the length of the wave path in corresponding areas. The antenna array approach usually use a circular antenna array to excite array elements with the same amplitude but different phases.

Compared to these methods, metasurfaces for generation of beams carrying OAM have advantages including low profile and simple EM wave control, i.e., the magnitude/phase/polarization of the EM waves can be manipulated simply by changing the shape, geometry, size, orientation and arrangement of the structures [17]. Reflective metasurfaces were used to generate single and double mode vortex beams in mircrowave [18,19,20,21] and terahertz regimes [22]. An active transparent metasurface was proposed for generating EM beams carrying OAM in the microwave frequency range [23]. However, these designs only focus on the OAM controlling of microwaves leaving the polarization state of the transmitted wave the same as that of the incidence. Simultaneously control the polarization and OAM of EM waves can enhance the performances of OAM beams in applications like radar imaging. Recently, metasurfaces using Geometric-Phase were applied for simultaneous OAM and spin angular momentum control [24,25,26]. These techniques impart a new degree of freedom to EM wave control and pave a way for future applications.

In this paper, multi-layered metasurfaces generating OAM beams with efficient linear polarization conversion were proposed. The patches on the outer sides of the designed metasurface receive and re-radiate the incident wave, respectively. The cross-polarized transmission is higher than −1 dB around 5.15 GHz with an extremely low co-polarized transmission below −35 dB. The transmission phase can be fully controlled by the length of the stripline in the middle layer. By arranging the metasurface unit cells according to desired phase distributions, the proposed metasurfaces can generate EM beams carrying different modes of OAM. This design method was demonstrated by both simulation and measurement.

This paper is organized as follows: Section 2 presents the design of the unit cells and the metasurfaces. In this section, detailed geometries of the unit cell are introduced, the characteristics of the unit cell are shown and the metasurface designs are presented. Section 3 presents the simulated and measured results of the metasurfaces, which verify the designs and show OAM generation with polarization conversion. In Section 4, the conclusions are drawn and the originality of this work is presented.

## 2. Design of the Metasurface

**Figure 1:**
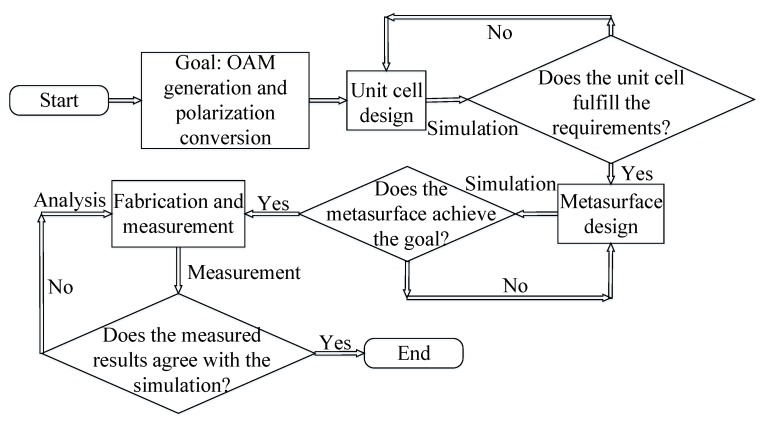
Flowchart illustrating the main goal and the adopted methodology of this paper.

**Figure 2:**
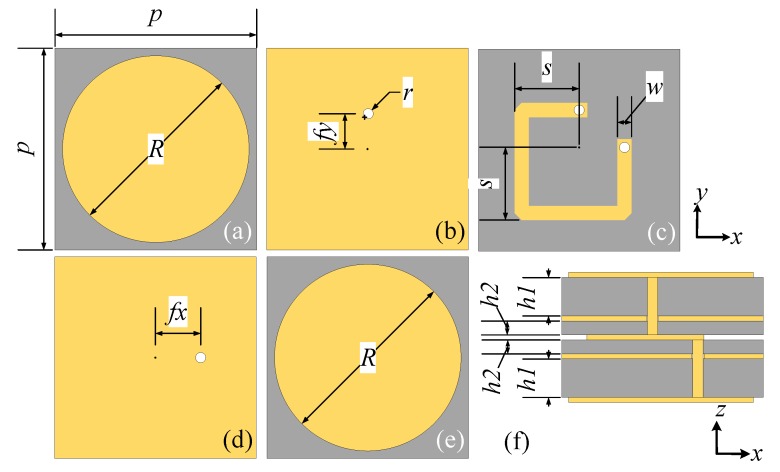
Geometry of the unit cell: (**a**) Top layer. (**b**) First ground layer. (**c**) Middle layer. (**d**) Second ground layer. (**e**) Bottom layer. (**f**) Side view.

A flowchart illustrating the main goal and the adopted methodology of this study is presented in Figure 1. The unit cell pattern of the proposed multi-layered laminated metasurface is depicted in Figure 2, where the gray parts represent the substrate Rogers 4350 B with $\epsilon_{r}$ = 3.48 and tanδ = 0.0037. The yellow parts represent the metal structures with a thickness of 0.035 mm. The top and bottom layers of the metasurface are circular patches which can couple or decouple the incident wave with a cross-polarization conversion. The middle layer of the metasurface is a stripline structure and is separated from the top and bottom layers by the first and second ground layers, respectively. Two vias connect the two ends of the stripline to the top and bottom layers, respectively. The geometric dimensions are *p* = 17.92 mm, *R* = 16.64 mm, *r* = 0.8 mm, *w* = 1.2 mm, $f_{x}$ = $f_{y}$ = 3 mm, $h_{1}$ = 1.524 mm and $h_{2}$ = 0.254 mm. The length of the stripline *S* varies from 1.66 mm to 8.49 mm to achieve a 360° transmission phase control.

The unit cell design takes its inspiration from the patch antenna. The top/bottom layer couples the y-polarized/x-polarized incident wave into the guided mode in the stripline structure and then, the bottom/top layer decouples the guided wave into the x-polarized/y-polarized free space propagation. It is by selecting the positions of the vias in orthogonal direction (e.g., in *x* and *y* directions), that the polarization of the transmitted wave is converted.

**Figure 3:**
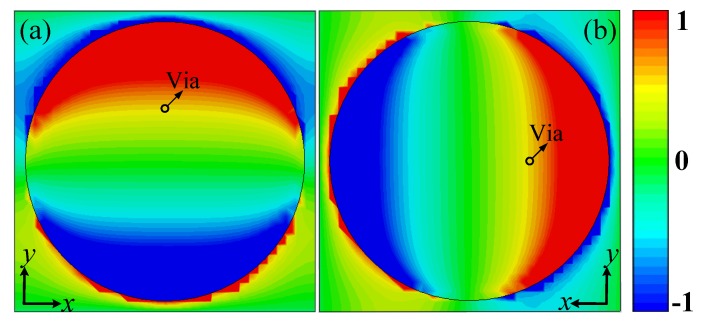
The simulated distributions of the electric field component perpendicular to the unit cell (i.e., E_*z*_): (**a**) Top layer. (**b**) Bottom layer.

The simulated distributions of the electric field component perpendicular to the unit cell (i.e., E_*z*_) on top and bottom layers are shown in Figure 3a,b, respectively. The incidences excite a y-polarized dipolar mode on the top layer, where the guided mode travels through the stripline structure to the bottom layer and excite a x-polarized dipolar mode, therefore converting the polarization of the transmitted waves. In addition, the guided mode experiences different phase delay when the length of the stripline varies. Also, the energy loss in the stripline structure is small and consistent regardless of its lengths. Therefore, the transmission phase can be controlled by the length of the stripline, which allows different phase distributions for different OAM beam-generating, while the transmission loss is small and stable. Notably, due to only the top and bottom layers resonate, this design has potentials to obtain low insert loss.

**Figure 4:**
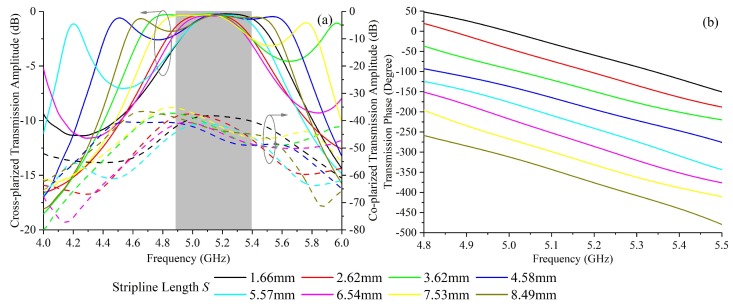
The simulated transmittance of the unit cell with different stripline lengths *S* (as in Figure 2c): (**a**) Amplitude. (**b**) Phase.

The unit cell models were simulated by the commercial software CST Microwave Studio (Version 2016, Computer Simulation Technology GmbH, Darmstadt, Germany) using periodic boundary in *x* and *y* directions. The simulated transmission amplitudes and phases are shown in Figure 4a,b, respectively. The transmission phase data at 4.8–5.5 GHz are given because, at other frequency ranges, the curves are confused and not of main concern in this paper. For a *y*-polarized incidence propagating along −z direction, the transmitted wave is *x*-polarized and the cross-polarized transmission amplitudes with different *S* are higher than −0.5 dB at 5.14 GHz, and from 4.9 GHz to 5.4 GHz, the transmittances are higher than −3 dB which indicates a 50% power efficiency, as shown in Figure 4a. The co-polarized transmittances are below −35 dB from 4.9 GHz to 5.4 GHz, indicating an extremely high polarization conversion efficiency, compared with [27,28]. At 5.14 GHz, the co-polarized transmittance is below −38.2 dB. Eight stripline lengths were selected with a transmission phase step of 45° to cover a 360° phase difference, as shown in Figure 4b. The selected stripline lengths are 1.656 mm, 2.62 mm, 3.62 mm, 4.58 mm, 5.57 mm, 6.54 mm, 7.53 mm and 8.49 mm.

**Figure 5:**
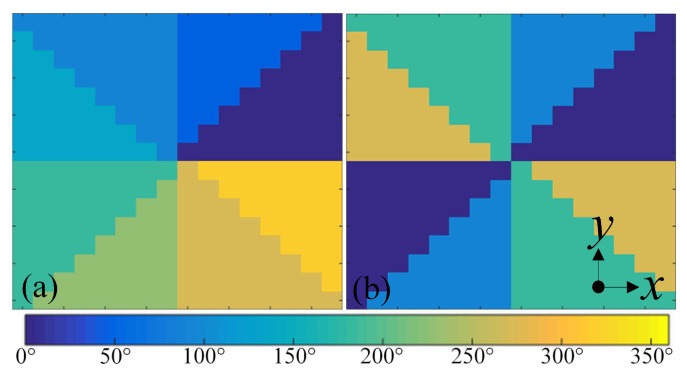
The front view of the transmission phase distribution schemes at 5.14 GHz for generating beams carrying OAM of different orders: (**a**) l=+1. (**b**) l=+2.

The helical wavefront of vortex beams can be expressed by the term exp(ilΦ), where Φ is the azimuthal angle and *l* is the topological charge. Therefore, EM beams carrying OAM with an order of *l* experiences an azimuthal phase change of ∣l∣×360°. The sign of the OAM order *l* defines the helicity of the vortex beam phase distribution. To generate vortex beam-carrying OAM of orders ±1 and ±2, two transmission phase distributions at 5.14 GHz with phase steps of 45° and 90°, respectively were designed and shown in Figure 5a,b, which depict the desired transmission phase with regard to different positions on metasurfaces. For wave propagating along −z and *z* directions, these two designs have opposite helicities and generate EM beams carrying OAM with orders of 1/2 and −1/−2, respectively.

**Figure 6:**
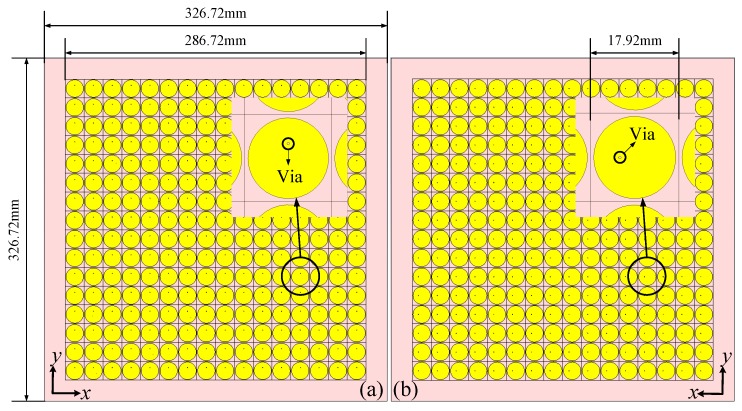
The simulation model of the proposed metasurface: (**a**) Front view. (**b**) Back view.

The two finite full structure models containing 16×16 unit cells are shown in Figure 6. The discretization of the metsurface is done according to the transmission phase distributions in Figure 5. The target frequency of the metasurfaces is at 5.14 GHz. It is worth pointing out that the potential applications for radar imaging can be in X/C/S band and the metasurface design can be easily tuned to other frequencies as well. The models were built up and simulated by CST Microwave studio with a Gaussian beam as excitation with a minimum beam radius of 100 mm on the metasurface. The average simulation time in a server with 256 GB memory and Intel Xeon E5 CPU is about 6 h. About 30 GB memory is used. Gaussian beam, compared with plane wave, reduces the slight amount of diffractions of the EM waves at the margins, while the phase profile of the transmitted beams are the same. Also, the margins of the metasurfaces have metal sheet in ground layers to further avoid diffractions. The used unit cells for realizing the desired transmission phase distribution designs in Figure 5a,b with phase steps of 45° and 90° respectively are selected from Figure 4b. Eight kinds of unit cell with stripline lengths of 1.656 mm, 2.62 mm, 3.62 mm, 4.58 mm, 5.57 mm, 6.54 mm, 7.53 mm and 8.49 mm are selected for Figure 5a while four kinds of unit cell with stripline lengths of 1.656 mm, 3.62 mm, 5.57 mm and 7.53 mm are selected for Figure 5b.

## 3. Results

**Figure 7:**
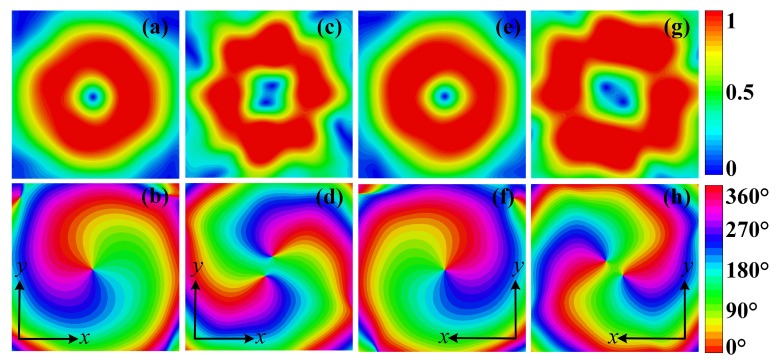
Simulated cross-polarized electric field distributions of the transmitted OAM carrying beams at a transverse plane 250 mm away from the metasurface: (**a**) Amplitude and (**b**) phase distributions for OAM order of l=+1. (**c**) Amplitude and (**d**) phase distributions for OAM order of l=+2. (**e**) Amplitude and (**f**) phase distributions for OAM order of l=−1. (**g**) Amplitude and (**h**) phase distributions for OAM order of l=−2.

The simulated transmitted electric field distributions at a transverse plane 250 mm away from the metasurface are depicted in Figure 7. Figure 7a–d,e–h show the transmitted electric field distributions with incidences propagating along −z direction with a *y*-polarization and along *z* direction with a *x*-polarization, respectively. Figure 7a,c,e,g show the normalized transmitted electric field amplitude distributions at 5.14 GHz with design schemes in Figure 5a,b, respectively. Amplitude nulls can be observed due to the phase singularity at the center of OAM carrying beams, and along with the donut-shaped field distribution verified the characteristic of the vortex beams.

The transmitted phase distributions are shown in Figure 7b,d,f,h. The phase accumulations along a full circular path around the beam null in Figure 7b,d are 2π and −2π, which indicates OAM orders of +1 and −1, respectively. Figure 7f,h depict 4π and −4π phase accumulations along a full circular path and therefore indicate OAM orders of +2 and −2. Thus, by using the proposed structure, the designed metasurfaces can simultaneously convert the polarization of the incident wave and generate vortex beams carrying OAM of four different orders, which has great potentials for radar imaging applications.

**Figure 8:**
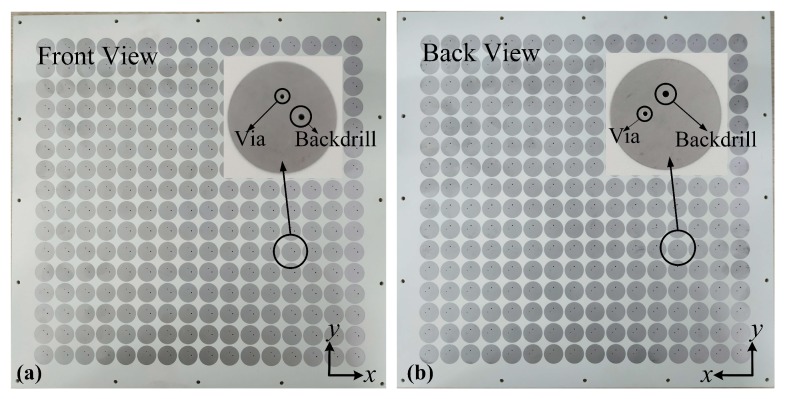
Photos of the fabricated metasurface: (**a**) Front view. (**b**) Back view.

The proposed metasurface was fabricated using PCB processing as shown in Figure 8. The overall size of the fabricated sample is 326.72 mm × 326.72 mm with a thickness of 4.35 mm. Vias connecting the middle layer to the top and bottom layers are fabricated by back drilling leaving two holes on the top and bottom layers of each unit cell. The back drill holes and prepregs have been considered in the simulations and have little influences on the metasurfaces performance.

**Figure 9:**
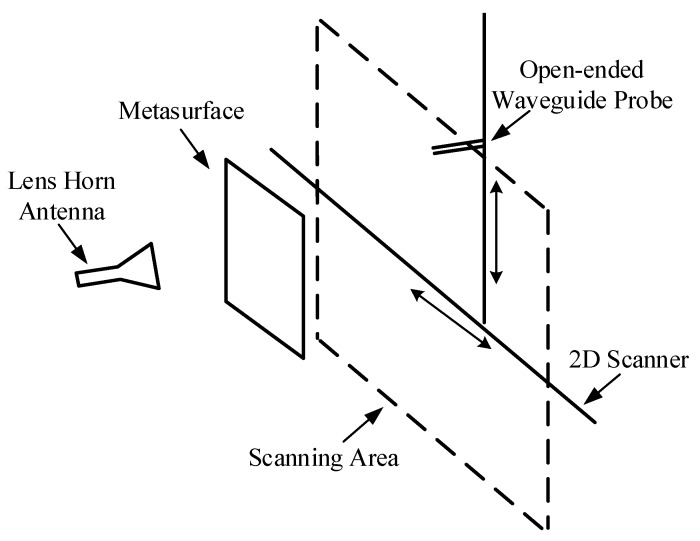
Schema depicting the measurement devices and settings.

The fabricated metasurfaces were measured using a vector network analyzer Agilent E8363b (Keysight Technologies, California, United States) and a two-dimensional near field scanning measurement system. The metasurface was placed between a lens horn antenna (used as the excitation) and a WR-229 open-ended rectangular waveguide probe (used for receiving the OAM carrying beams). The measurement was conducted in the anechoic chamber. The response calibration was used to eliminate the effect of external noise during the measurement. The polarization conversion was confirmed by receiving and analyzing the co-polarization and cross-polarization components of the EM waves, which was realized by rotating the open-ended rectangular waveguide probe. A schema of the measurement devices and settings is depicted in Figure 9.

**Figure 10:**
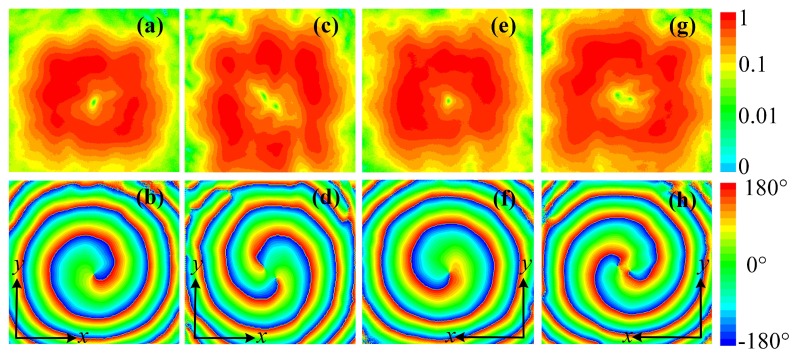
Measured cross-polarized electric field distributions of the transmitted OAM carrying beams at a transverse plane 250 mm away from the metasurface: (**a**) Amplitude and (**b**) phase distributions for OAM order of l=+1. (**c**) Amplitude and (**d**) phase distributions for OAM order of l=+2. (**e**) Amplitude and (**f**) phase distributions for OAM order of l=−1. (**g**) Amplitude and (**h**) phase distributions for OAM order of l=−2.

The measured amplitude and phase distributions of the cross-polarized transmitted electric field at a transverse plane 250 mm away from the metasurface are shown in Figure 10. Figure 10a–d show the amplitudes and phases of the *x*-polarized transmitted wave with a *y*-polarized excitation propagating along −z direction. Figure 10a,c depict an amplitude null at the field center. Figure 10b,d show phase accumulations of 2π and 4π along a full circular path, indicating OAM orders of +1 and +2, respectively. Figure 10e–h show the amplitudes and phases of the *y*-polarized transmitted wave with an *x*-polarized excitation propagating along *z* direction. The amplitude distributions shown in Figure 10e,g show amplitude nulls level at 0.01 compared to the maximum value. Due to the deviations in fabrication and measurement, the perfect offset of amplitude at the center may be compromised. Still, −20 dB nulls level is satisfying [29]. The phase distributions in Figure 10f,h show −2π and −4π phase accumulations along a full circular path, indicating OAM order of −1 and −2, respectively.

**Figure 11:**
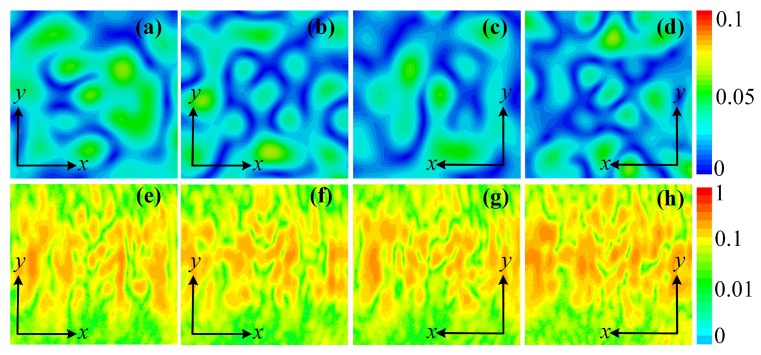
Simulated co-polarized electric fields amplitude distributions for different OAM orders: (**a**) l=+1. (**b**) l=+2. (**c**) l=−1. (**d**) l=−2. Measured co-polarized electric fields amplitude distributions for different OAM orders: (**e**) l=+1. (**f**) l=+2. (**g**) l=−1. (**h**) l=−2.

The simulated and measured amplitudes of the co-polarized transmitted wave are shown in Figure 11a,b, respectively. For each condition, the co-polarized transmissions are randomly distributed with a low amplitude. In the simulation results, the co-polarized electric fields amplitude level is lower than 0.06, while the measured results show co-polarization level lower than 0.15. The discrepancy between simulation and measurement comes from fabrication deviations and the slight amount of diffracted EM waves. However, compared with the cross-polarization level, the co-polarization level is low and does not affect the generated cross-polarized vortex beams. The co-polarization level can be enhanced if absorbers are placed around the metasurface.

## 4. Conclusions

In conclusion, two polarization conversion metasurfaces generating four different orders of OAM carrying beams (l=+1,l=+2,l=−1 and l=−2) were designed and fabricated. The simulation and measurement results are in good agreement with each other. The multi-layered unit cells we proposed realize full phase control, low transmission loss, high polarization conversion efficiency and can be easily tuned to any frequencies of interest. By manipulating the transmission phase distributions on the metasurface aperture, the transmitted beams can carry OAM of four different orders, which has potentials for super resolution imaging. In addition, the polarizations of the transmitted waves were efficiently converted, which may further enhance the performances in applications, for example, imaging polarization dependent objects.
